# Single-Cell Untargeted Lipidomics Using Liquid Chromatography and Data-Dependent Acquisition after Live Cell Selection

**DOI:** 10.1021/acs.analchem.3c05677

**Published:** 2024-04-23

**Authors:** Johanna von Gerichten, Kyle D. G. Saunders, Anastasia Kontiza, Carla F. Newman, George Mayson, Dany J. V. Beste, Eirini Velliou, Anthony D. Whetton, Melanie J. Bailey

**Affiliations:** † School of Chemistry and Chemical Engineering, Faculty of Engineering and Physical Sciences, University of Surrey, GU2 7XH Guildford, U.K.; ‡ Cellular Imaging and Dynamics, GlaxoSmithKline, Stevenage SG1 2NY, U.K.; § School of Bioscience, Faculty of Health and Medical Sciences, University of Surrey, GU2 7XH Guildford, U.K.; ∥ Centre for 3D Models of Health and Disease, University College London, Division of Surgery and Interventional Science, London W1W 7TY, U.K.; ⊥ vHive, School of Veterinary Medicine, School of Biosciences and Medicine, University of Surrey, Guildford GU2 7XH, U.K.

## Abstract

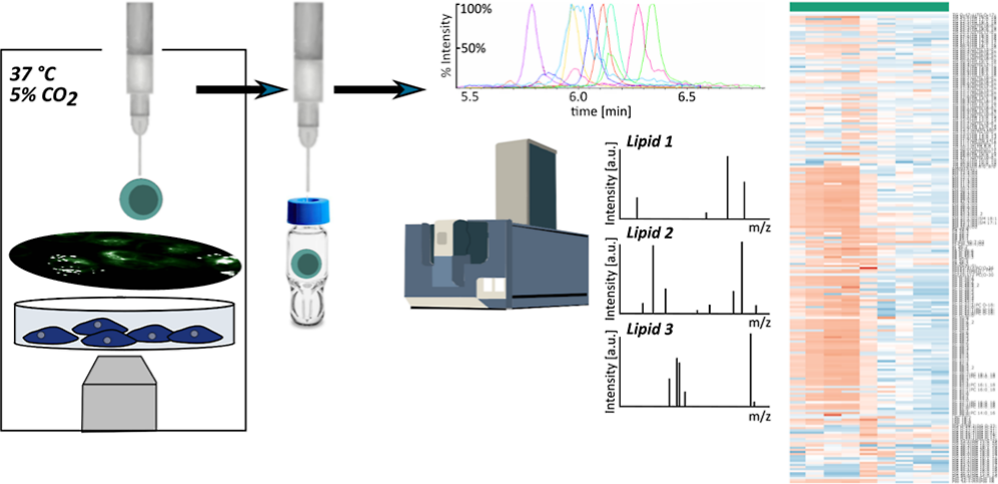

We report the development and validation of an untargeted single-cell lipidomics method based on microflow chromatography coupled to a data-dependent mass spectrometry method for fragmentation-based identification of lipids. Given the absence of single-cell lipid standards, we show how the methodology should be optimized and validated using a dilute cell extract. The methodology is applied to dilute pancreatic cancer and macrophage cell extracts and standards to demonstrate the sensitivity requirements for confident assignment of lipids and classification of the cell type at the single-cell level. The method is then coupled to a system that can provide automated sampling of live, single cells into capillaries under microscope observation. This workflow retains the spatial information and morphology of cells during sampling and highlights the heterogeneity in lipid profiles observed at the single-cell level. The workflow is applied to show changes in single-cell lipid profiles as a response to oxidative stress, coinciding with expanded lipid droplets. This demonstrates that the workflow is sufficiently sensitive to observing changes in lipid profiles in response to a biological stimulus. Understanding how lipids vary in single cells will inform future research into a multitude of biological processes as lipids play important roles in structural, biophysical, energy storage, and signaling functions.

## Introduction

Lipids are an essential part of a cell’s biomolecular pool and play important roles in a myriad of complex biological processes with biophysical, energy storage, and signaling functions (reviewed in^1−3^). Recent studies demonstrate that analyzing lipids at the single-cell level not only reveals significant variation in lipid profiles between different cells^4−7^ but is also capable of observing putative response to stimuli, such as treatment with drugs or exogenous fatty acids.^8−10^ Cancer cells are highly heterogeneous and very diverse in their phenotype which makes them hard to extinguish, but the analysis of single cells and the characterization of the heterogeneity could provide new treatment strategies.^11^ Pancreatic ductal adenocarcinoma is the most common pancreatic cancer with the poorest prognosis for pancreatic diseases.^12^ However, the analysis of a single cell presents several unique challenges due to the low abundance of analytes present in one cell. New methods are fast emerging as many of the technical challenges for handling and analyzing single cells are addressed (reviewed in^13−15^).

A first challenge for single-cell lipidomics is sampling living single cells as even minor changes to the environment of a cell can lead to stress reactions that alter the lipidome. There are several commercially available techniques for single-cell handling that involve cells being in a suspension for microfluidics or droplet-based devices (reviewed in^16^). However, to construct accurate maps of lipid processes in individual cells, the selection of living cells from their native microenvironment is crucial and must take account of the fact that many normal cells adhere to a substratum (e.g., extracellular matrix). Moreover, these approaches lose spatial contextualization of the cells.

Capillary sampling involves the microscopy-based detection of adherent cells and sampling via glass capillaries under negative pressure. It carries the advantage of preserving the spatial location of the cells, critical for answering questions surrounding, for example, cell communication (i.e., bystander effects) and has been successfully used to detect drug levels and tentative lipid profiles in single cells.^13,17^ However, to date, this manual sampling approach has been performed only under uncontrolled environmental conditions.

A second challenge for single-cell lipidomics is the sensitivity. Capillary sampling has typically been coupled to a static nanospray for analysis. However, this carries the disadvantage of ionization suppression and lacks automation, in terms of both data collection and analysis. For untargeted lipidomics analysis of bulk samples, fragmentation-based (MS^2^) identification of lipids via liquid chromatography tandem mass spectrometry (LC MS/MS) using data-dependent acquisition (DDA) has become the gold standard and has the advantage of automation.^18^ The use of a chromatographic system results in a reduction of ionization suppression, increases sensitivity to minor lipid species, and expands the lipid coverage. DDA MS improves the confidence in lipid identification by MS^2^-based database confirmation, facilitating untargeted analysis.^19−21^ The integration of capillary sampling with a bulk-lipidomics approach is therefore highly attractive for untargeted single-cell lipidomics, but a method capable of generating MS^2^ spectra remains challenging due to the very low lipid mass present in single cells.

Here, we demonstrate (I) the development of a single-cell DDA lipidomics method with microflow chromatography to identify lipids with high confidence based on MS^2^. In the absence of single-cell standards, the methodology is optimized and validated using diluted bulk cell extract. (II) We coupled DDA lipidomics to an automated cell selection system to provide a workflow for high confidence lipid identification in single cells. Finally, in (III), we used the workflow to combine live cell imaging of pancreatic cancer cells with single-cell lipidomics. An increase in the lipid droplet size was detected based on fluorescence microscopy and found to correlate with the higher abundance of neutral DG and TG lipids measured with DDA lipidomics.

## Experimental Section

### Cell Culture

Human pancreatic adenocarcinoma cells PANC-1 and ASPC-1 (Merck, UK) were cultured in DMEM glucose (Sigma-Aldrich, UK, cat no. 21969035) with 10% (v/v) fetal bovine serum (Fisher Scientific, UK, cat no. 11550356), 1% penicillin/streptomycin (Fisher Scientific, UK, cat no. 15140122), and 2 mM l-glutamine (Sigma-Aldrich, UK, cat no. 25030024). Cells were kept at 37 °C with 21% O_2_ and 5% CO_2_. Cell culture media was replaced on alternate days, and cells were passaged approximately once a week when confluency reached approximately 80%. 48 h prior to single-cell sampling, 200,000 cells were seeded into a 3.5 cm Nunc Glass Bottom Dish (150682 Thermo, UK). 2 mL of cell culture media (no cells) was aliquoted into a cell culture dish to serve as negative control. Cells were washed twice with 37 °C FBS-free culture medium and left in 2 mL of FBS-free culture medium for cell sampling. To mimic oxidative stress, PANC-1 cells were treated with 0.4 mM hydrogen peroxide (H_2_O_2_, 3 wt % solution in water; Acros Organics) for 1 h at 37 °C, H_2_O_2_ was added directly to the cell culture medium. Both untreated and treated cells were washed twice with 37 °C FBS-free culture medium and left in 2 mL of FBS-free culture medium for cell sampling. No necrotic cell death was observed in either population.

The THP-1 human monocytic cell line was obtained from ATCC TIB-202. Cells were grown in RPMI 1640 medium supplemented with 0.2% glucose, 0.2% sodium bicarbonate, and 10% heat-inactivated fetal-calf serum (FCS) (Sigma). THP-1 cells were differentiated with 50 nm phorbol-12-myristate-13-acetate for 72 h at 37 °C, 5% CO_2_, and 95% humidity. Cells were washed with PBS supplemented with 0.49 mM Mg^2+^ and 0.68 mM Ca^2+^ (PBS^+^) and 1% FCS. For sampling, 1 × 10^6^ THP-1 cells from the suspension were plated onto a 27 mm glass culture dish (Thermo Scientific) and a Nunc 6 well plate (Thermo Fisher), in preparation for single-cell sampling and bulk lipid extraction, respectively.

### Lipid Extract from Bulk Cells

Cells were put into the suspension phase using trypsin and centrifuged at 300×*g* for 5 min and then washed with ice-cold Dulbecco’s phosphate-buffered saline (Sigma-Aldrich, UK). The cell pellet was suspended in 1 mL of water and flash frozen in liquid nitrogen. The cell pellet was then subjected to two cycles of freeze–thaw (37 °C for 10 min, liquid nitrogen for 30 s) to aid cell lysis. Lipids were extracted by a modified Folch extraction using a chilled solution of methanol/chloroform (1:2 v/v) supplemented with 0.01% butylated hydroxytoluene (BHT, Fisher Scientific, UK, cat no. 11482888) to prevent lipid oxidation according to the modified protocol described by Zhang et al.^22^ Both methanol and ethanol were of Optima LC–MS grade and were purchased from Fisher Scientific. The bottom layer of the extraction was taken and dried down under nitrogen, stored at −80 °C, and reconstituted on the day of analysis in the starting mobile phase 70:30 A/B containing 16 ng/mL EquiSPLASH (Avanti Polar Lipids, cat no. 330731). The bulk lipid extract from THP-1 macrophages was diluted to 50 cells and single cells per 5 μL using mobile phase 70:30 A/B containing EquiSPLASH (16 ng/mL).

### Automated Cell Sampling with the Yokogawa SS2000 Single Cellome System

Cells were cultured for 72 h in media depending on cell type within glass culture dishes and washed with warm FBS-free media before capillary sampling. Cells were kept in fresh FBS-free media. The 35 mm culture dish was introduced to the Yokogawa SS2000 Single Cellome System, where living single cells were sampled using 10 μm capillaries (Yokogawa). Single cells were manually selected at random in the direct mode with the following pressures: presampling 6 psi, sampling 14 psi, and postsampling 3 psi. The cells were sampled with a single pick and held for 200 ms. The tips were immediately frozen after cell sampling using dry ice. Single cells were transferred and stored at −80 °C for future use. Cells were kept at 37 °C with 5% CO_2_ during sampling.

Cells were transferred from the capillary into total recovery LC–MS vials (Waters) by backfilling the capillaries with 5 μL of lysis solvent that consisted of starting mobile phase 70:30 A/B spiked with EquiSPLASH (Avanti Polar Lipids, cat no. 330731; 16 ng/mL) and using a gas syringe with a Luer lock adapter to elute the solution into the vial, as described previously.^13^ LC–MS/MS (DDA lipidomics) analysis was performed on the same day of elution, and the total 5 μL volume was injected into the column.

### Nile Red Staining and Lipid Droplet Analysis

PANC-1 cells, untreated and H_2_O_2_ treated, were stained for lipid droplets by adding 2 mM Nile Red^23^ (9-(Diethylamino)-5*H*-benzo[α]phenoxazin-5-one, Sigma-Aldrich, UK) solution to the media for 15 min at 37 °C and were washed twice with 37 °C FBS-free culture medium and left in 2 mL of FBS-free culture medium for cell sampling. Images were produced using a UPLXAPO 40*x*/0.95 dry (WD 0.18) objective and 8 μm z-stack projection for both brightfield and fluorescence (Ex 488 nm/Em 617 ± 73 nm) using the Yokogawa SS2000. ImageJ was used to measure individual cell diameter, mean gray value of the fluorescent signal, and area with the ROI manager, multi measure tools, and the particle size analyzer.^24^

### Lipidomics Analysis—LC–MS/MS

Single cells were thawed and capillary-eluted on the day of analysis with 70:30 mobile phase A/B containing 16 ng/mL EquiSPLASH. Lipids were detected by data-dependent analysis (DDA) using an Acquity M-class (Waters) coupled to a 7600 ZenoTOF system (Sciex). Positive ESI parameters were as follows: 4500 V spray voltage, 80 V declustering potential, 350 °C source temperature; *m*/*z* range 150–900, collision energy MS^1^ 12 V, and CID 35 V. The top 30 fragment ions were detected with 0.2 ms acquisition time and without dynamic background correction.

The chromatography method was adopted from previous work and modified for microflow.^13^ Briefly, lipids were separated using a C18 column (Luna Omega 3 μm Polar C18 100 Å 50 × 0.3 mm; Phenomenex) at 40 °C and a flow rate of 8 μL/min. The mobile phases were all made from Optima LC–MS grade solvents purchased from Fisher Scientific and were A 60:40 (v/v) acetonitrile/water and B 85:10:5 (v/v) isopropanol/water/acetonitrile, both containing 0.2% (v/v) formic acid (Fisher Chemical Optima LC/MS grade) and 10 mM ammonium formate (99%; Acros Organics). The LC gradient decreased from 60% A at 0.5 min to 1% A at 4.5 min, stayed isocratic for 2 min, increased from 20% A at 6.5 min to 60% A at 11 min, and stayed isocratic for 4 min. Data were acquired using Sciex OS (Version 3.0.339).

### Data Analysis

MS-Dial (ver.4.9.221218) was used to process the raw LC–MS/MS data by using a mass tolerance of 0.01 Da for MS^1^ and 0.025 Da for MS^2^ to collect the data. Peak detection was performed with a minimum peak height amplitude of 300 and a mass slice width of 0.1 Da. Smoothing was carried out using a linear weighed moving average with a 2 scan smoothing level and 5 scan minimum peak width. Peak identification was run with a mass tolerance of 0.01 Da for MS^1^ and 0.025 Da for MS^2^, with an 80% identification score cutoff. Adducts of [M + H]^+^, [M + NH_4_]^+^, and [M + H – H_2_O]^+^ were allowed. The alignment parameters were 0.1 min retention time tolerance and 0.025 MS^1^ tolerance; peaks with less than 10% area count (relative to the maximum area count detected) were removed. Each detected lipid with signals in less than 60% of samples within a group was excluded, and gap filling by compulsion was disabled. As a reference file for alignment, the data from a bulk cell extract of 28 cells/μL was used. Semiquantitative value assignment was performed manually in Excel (Microsoft). Values were normalized to the number of cells in the cell culture for bulk extracts and background-corrected for organic solvents (bulk) or cell media (single-cells). The internal standard EquiSPLASH was used to calculate lipid concentrations as pmol/mL. Only the following lipid classes covered by the internal standard are reported: LPC, Cer, MG, DG, PC, PE, PI, PG, PS, SM, and TG including the ether-form of these lipids. Zero values were removed. Multivariate analysis was carried out using MetaboAnalyst 5.0.^25^ For relative abundance value assignments, the data were log transformed as experiment/control and autoscaled (mean-centered and divided by the standard deviation of each variable) before partial least-squares–discriminant analysis (PLS-DA). Variable importance in projection (VIP) was calculated for each lipid. GraphPad Prism version 8.4.3 for Windows (GraphPad Software, San Diego, California USA) was used for one-way ANOVA and *t*-test. The calculations for the limit of detection (LoD) and limit of quantification (LoQ) are described in the supplement.

## Results and Discussion

Here, we developed a method for single-cell DDA lipidomics based on microflow liquid chromatography with MS^2^ DDA showing sensitivity for the widely used database identification of lipids in single cells. Furthermore, we demonstrate fluorescent live cell imaging coupled to the single-cell lipidomics method and thereby the correlation of cellular events with omics techniques, as shown through the example of expanding lipid droplets and changes in the lipid composition.

### Validation of Single-Cell DDA Lipidomics with “1 Cell Equivalent” Injections

**Figure 1:**
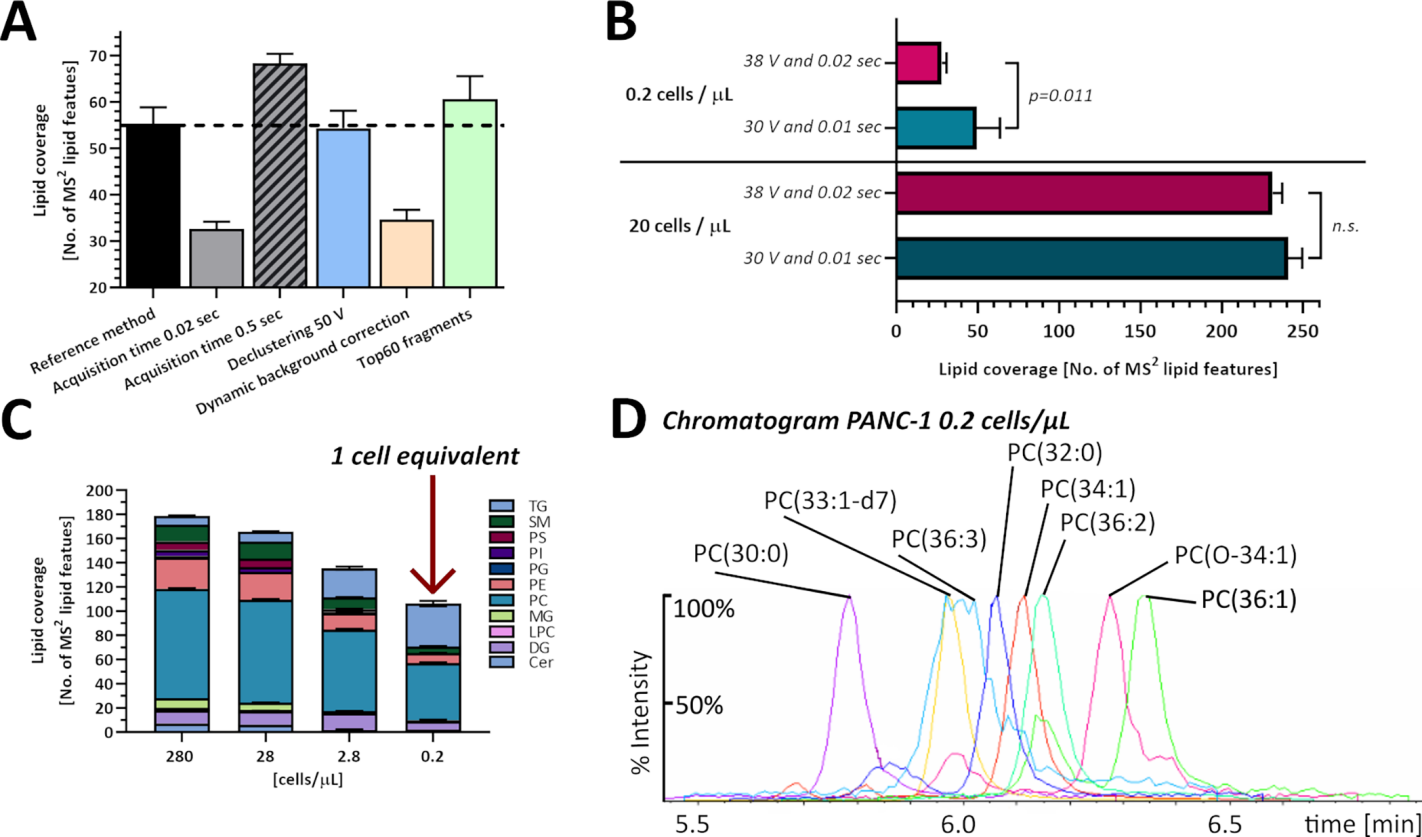
DDA lipidomics method validation with diluted lipid extracts. (A) DDA lipidomic parameter optimization using diluted porcine brain polar lipid extracts (*n* = 3). (B) Change in lipid coverage due to different DDA lipidomics parameters (V collision energy and sec MS^2^ accumulation time) using the diluted PANC-1 extract for method development compared to 1 cell equivalent PANC-1 extract (*n* = 3). (C) Lipid coverage in PANC-1 dilution series (*n* = 3). (D) Chromatogram of PC species from PANC-1 extract at 1 cell equivalent injection (0.2 cells/μL), with all peaks set to 100%.

Single-cell mass spectrometry, especially lipidomics, is in its infancy, and therefore relevant standards do not exist. Isolated single cells are not suitable for method parameter optimization due to their heterogeneity. A diluted commercially available lipid extract was used for initial method optimization, as shown in Figure 1 A. DDA parameters were optimized for maximum lipid coverage with a dilute porcine brain polar lipid extract (Avanti) for collision energy (Figure S1), acquisition time, declustering potential, dynamic background correction, and amount of fragment ions (Figure 1A).

Our data highlight that dilution can be an important parameter in method optimization. Figure 1B shows that changing the collision energy (30/38 V) and accumulation time (0.01/0.02 s) has no significant influence on the lipid coverage when using a PANC-1 cell extract diluted to 20 cells/μL (equivalent to 100 cells/injection). In contrast, diluting a PANC-1 extract to 0.2 cells/μL (equivalent to 1 cell/injection) results in a significant difference in lipid coverage. Therefore, it is noteworthy that method optimization based on a cell extract without sufficient dilution can result in suboptimal conditions for single cells. We therefore propose the use of a diluted cell extract (“1 cell equivalent”) as part of the workflow for single-cell LC–MS/MS method development and validation. The final method parameters are as described in the Methods section.

To provide a guideline on the method sensitivity needed for lipidomics single-cell mass spectrometry, we assessed the LoQ and LoD for the lipid classes discussed in this paper (method in eq. S1). To do this, the internal standard (EquiSPLASH) was added to PANC-1 cell extract (14 cells/μL) in five concentrations from 8 to 0.5 ng/mL and analyzed with DDA lipidomics. The results are shown in Table 1; calibration curves and chromatogram are shown in Figures S2 and S3.

**Table 1:** LoD, LoQ, and Coefficient of Determination (*R*^2^) Calculated from a 5-Point Calibration Curve for Avanti EquiSPLASH Standard in 14 Cells/μL PANC-1 Cell Extract Matrix (*n* = 3; Values are Mean)

EquiSPLASH in PANC-1 extract	*m*/*z*	*R*^2^	LOD [nM]	LOQ [nM]
PC 15:0–18:1(d7)	753.613	0.995	0.71	2.15
PE 15:0–18:1(d7)	711.566	0.976	1.66	5.02
PG 15:0–18:1(d7)	759.588	0.998	0.50	1.51
PI 15:0–18:1(d7)	847.604	0.988	1.01	3.07
PS 15:0–18:1(d7)	755.556	0.937	2.59	7.86
TG 15:0–18:1–15:0(d7)	829.799	0.928	3.56	10.79
DG 15:0–18:1(d7)	605.584	0.997	1.13	3.43
MG 15:0–18:1(d7)	381.370	0.978	1.54	4.68
SM 18:1–18:1(d9)	738.647	0.992	0.84	2.54
Cer C15(d7)	531.548	0.993	1.18	3.58

To ensure robust analysis of low volume (5 μL) single-cell samples, we used the EquiSPLASH standard as quality control for low volume injection (5 μL of a total of 5 μL) within the batch and for high volumes (5 μL of a total of 100 μL) between the batches. The intra-assay variation with the low volume injection is 20% ± 3 and the interassay variation is 8% ± 2, except for TG (55% CV) (Table S1).

Sensitivity is a challenge for single-cell analysis, so to minimize dilution effects, the method uses a 5 μL injection from a total 5 μL volume in the vial. To explore the efficiency of this process, we evaluated the recovery of each individual lipid from the EquiSPLASH standard when injecting a 5 μL sample from a total 5 μL volume in the vial (Figure S4 left). Losses due to injection were between 15 and 39% of the total EquiSPLASH lipid material. There are several elements that could lead to the relatively high amount of sample loss, one being the injection loop of the autosampler. According to the manufacturers “partial loop” mode that was used here, samples were neither used for overloading the loop nor for prewash/conditioning of the loop, so there should not be any loss due to the injection mode. We tested a 10 μL injection loop in comparison to the 5 μL injection loop used for the above results (Figure S4 on the right). The loss of lipid material did not significantly improve (14–32%), indicating that the loop is not the cause of sample loss. Other possibilities for future work to explore are the vial type, needle height, and draw speed, which may enhance the recovery.

To evaluate the lipid coverage of the DDA lipidomics method at low cell count, the PANC-1 cell extract was analyzed with concentrations of 280, 28, 2.8, and 0.2 cells/μL (Figure 1C). In this system, 0.2 cells/μL of bulk cell extract using a 5 μL injection into the LC–MS/MS system is equivalent to the amount of lipids from a single-cell, and we refer to this as “1 cell equivalent”.

Lipid features with a confirmed fragmentation mass spectrum (MS^2^) were counted, and the total number was used as measure for lipid coverage. The coverage as number of lipid features detected decreased from a total of 180 ± 1 lipid features at 280 cells/μL down to 135 ± 2 lipid features at “1 cell equivalent” (0.2 cells/μL). 11 lipid classes included in the internal standard were used for coverage calculations (the list is given in Table S2). At PANC-1 concentrations of 2.8 cells/μL and below, lipids from the following classes PS, PI, PG, MG, LPC, and Cer could no longer be detected. Lipids identified in a PANC-1 “1 cell equivalent” are mainly DGs, TGs, and PCs, with a small number of PEs. Figure 1D shows a chromatogram from a PANC-1 “1 cell equivalent” extract of various PC species with the final method parameters. PC retention time changing with increasing chain length and grade of saturation is shown in Figure S5A.

To demonstrate the method’s relevance for biological applications at 1 cell equivalent injections, we tested the diluted cell extract from different cell lines: three cell lines of slightly different diameter PANC-1 (∼20 μm), ASPC-1 (∼15 μm), and THP-1 (∼10 μm).^26^Figure S5B–D shows that the DDA lipidomics method is sufficiently sensitive to see differences between cell types at 1 cell equivalent.

### Proof-of-Concept DDA Lipidomics on Capillary Sampled, Living Single-Cells versus “1 Cell Equivalent” Injections

**Figure 2:**
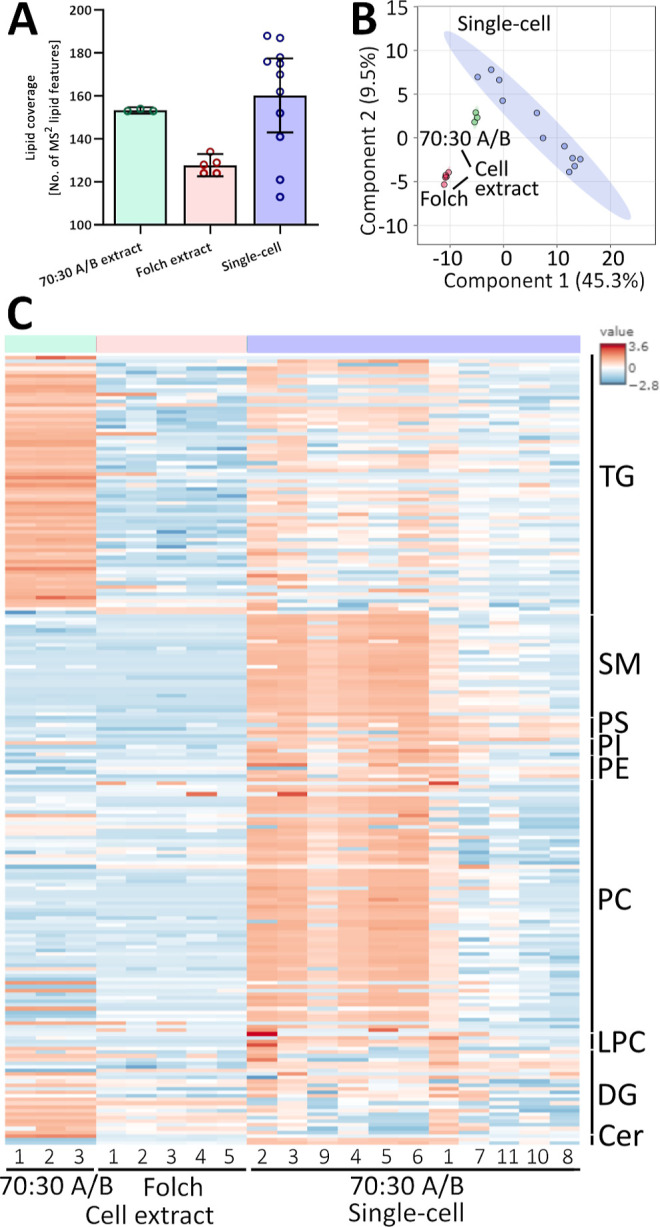
DDA lipidomics of PANC-1 living single cells in comparison to 1 cell equivalent injection (0.2 cells/μL) with two different extraction solvents. (A) Lipid coverage (*n* = 5 for Folch extracts, *n* = 3 for 70:30 mobile phase A/B extracts, and *n* = 11 for single cells; values are mean ± 95%CI); (B) PLS-DA score plot for 1 cell equivalent injections and single cells. The three groups are significantly separated (*R*^2^ = 0.988, *Q*^2^ = 0.944). (C) Heatmap for the individual lipid profiles from 1 cell equivalent injections and single cells (log transformed and autoscaled).

The developed method was used to analyze living pancreatic cancer (PANC-1) single cells that were capillary sampled. The cells were randomly selected in microcapillaries, immediately frozen, eluted into LC–MS vials with lysis solvent [mobile phase A/B 70:30 (w/w)] and used for DDA lipidomics analysis. Figure 2A shows the lipid coverage for 11 individually analyzed PANC-1 single-cells with 160 ± 8 lipids. This is similar to previously reported measurements of bulk PANC-1 cells.^22,27,28^

Referring to Table 1, the limit of detection for PC (33:1-d7) was 3.55 fmol. Calculating the theoretical concentration of PC (34:1) in a single cell based on an average from PANC-1 cells at 28 cells/μL (119 pmol/mL; *n* = 5) results in a theoretical concentration of 4.24 fmol of PC (34:1) in one cell. We detected concentrations between 12 and 1167 fmol PC 34:1 in single PANC-1 cells, therefore being in the range of expected concentrations, as well as over the limit of detection, supporting these observations.

Figure 2A compares the lipid coverage for PANC-1 single cells to a “1 cell equivalent”, extracted either with a modified Folch method or with mobile phase A/B 70:30 (w/w). The single cells were lysed in the same solvent system as that of the mobile phase A/B 70:30 (w/w) cell extract. Figure 2B shows the corresponding PLS-DA, and Figure 2C shows a heatmap of the same data.

The PLS-DA shows that the three groups are significantly separated, and the heatmap shows marked differences between single cells and cell extract. However, both the PLS-DA and heatmap indicate greater similarity in lipid profiles for single cells and the cell extract when the same solvent system is used. The limited overlap between the cell extract and single cells highlights the challenge in verifying single-cell data through analysis of a dilute cell extract. These data should be of particular interest to groups who use high-resolution mass spectrometry with MS^1^ data only for single-cell analysis (for example, those using MALDI or nanospray ionization) as it highlights the challenge of collecting a suitable “pooled QC” for MS^2^ spectral acquisition.

In all plots, the low variance of the cell extract reflects an averaged lipid signal from a heterogeneous cell culture, whereby in contrast, heterogeneity of individual cells is shown by the high variance between the individual single cells. The question of how many single cells are needed for significant biological challenges is still unanswered and will be a challenge in itself for future work.

### Biological Application of Single-Cell Lipidomics–Separation of Different Cell Types and Identification of Lipid Acyl Chains

**Figure 3:**
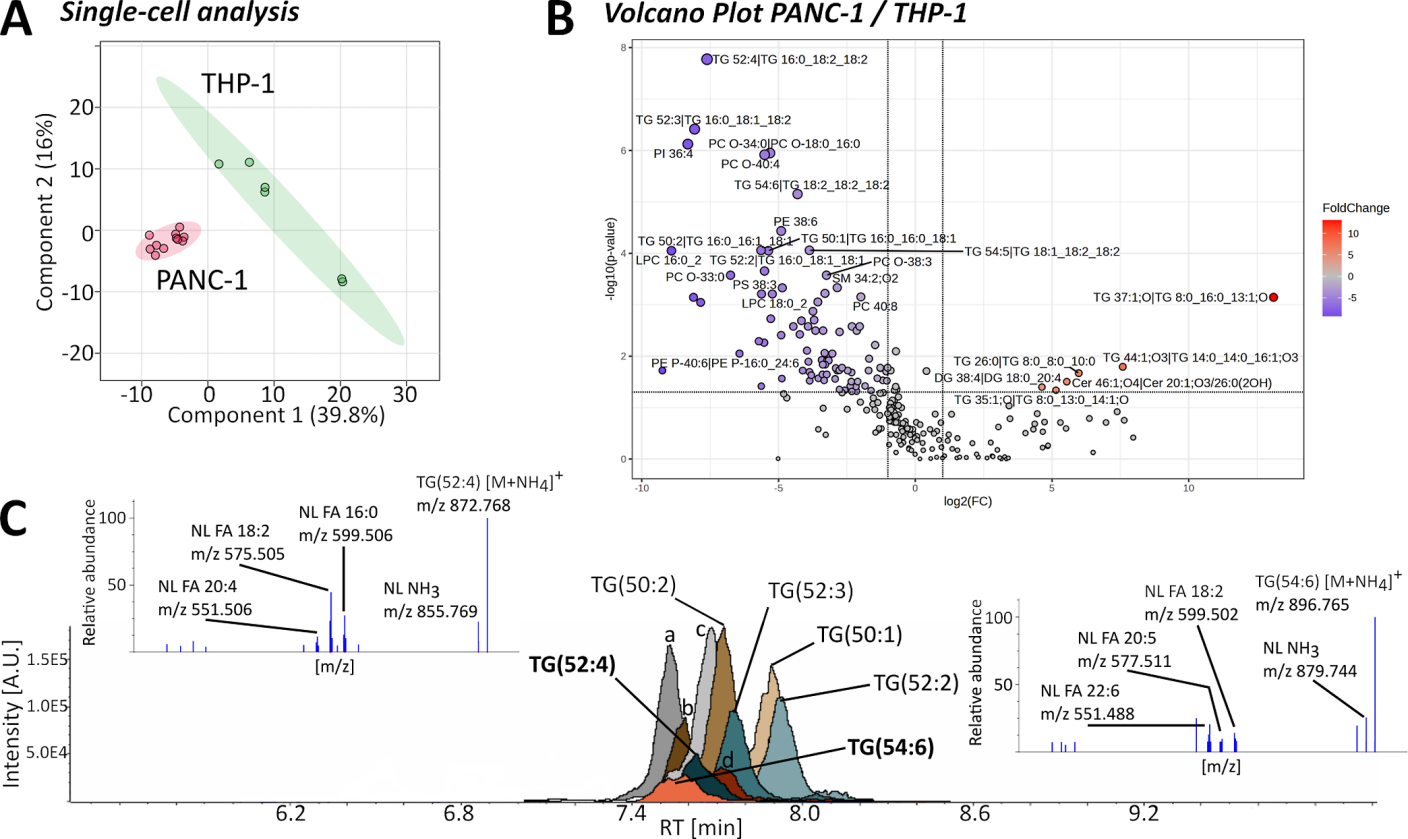
Separation of PANC-1 and THP-1 cell lines based on DDA lipidomics of single cells. (A) PLS-DA showing significant separation of two groups. (B) Volcano plot showing 151 not significant, 6 upregulated, and 89 downregulated lipids of the 246 MS^2^ identified lipids (PANC-1/THP-1). (C) Chromatogram with fragment spectrum of selected TG lipids from a single PANC-1 cell; (a) TG(48:2), (b) TG(50:3), (c) TG(48:1), and (d) TG(54:5) (THP-1 *n* = 6, PANC-1 *n* = 11). NL = neutral loss; FA = fatty acid.

To demonstrate the capability of the developed DDA lipidomics method for biological applications, we sampled living single cells from two different cell cultures (PANC-1 and THP-1) and analyzed the lipid profiles (Figure 3). PLS-DA showed the two groups separated significantly (*R*^2^ = 0.9812, *Q*^2^ = 0.9031; Figure 3A). Figure 3B shows the volcano plot for PANC-1/THP-1 with a total of 246 identified lipids. Of the total lipids, 61.4% are detected with similar concentrations in the two cell lines. The top 15 lipids from the VIP plot of the PLS-DA contain mostly TGs (7), with only four PCs and one PI, PE, LPC and SM, respectively; all of the VIP lipids were detected in higher abundance in the PANC-1 single-cells compared to the THP-1 cells (Figure S6 left). PANC-1 is a pancreatic cancer cell line, and pancreatic cancers have been shown to be surrounded by infiltrating immune cells such as macrophages (differentiated THP-1). For example, it has been shown that isolated PANC-1 exosomes induce a pro-tumoral or immunosuppressive phenotype (M2) in THP-1 macrophages, but little is known about the lipids that play a role in this cell-to-cell communication.^29^ Co-culturing of, for example, PANC-1 and THP-1 cells combined with capillary selection of single cells could help identify biomarker lipids for cell-to-cell communication with the workflow presented. Figure 3C shows a chromatogram from a single PANC-1 cell for various detected TG species as well as the MS^2^ mass spectra for TG(52:4) and TG(54:6). The chromatography method presented here is relatively short (15 min) and allows for chromatographic separation of most TGs (Figure S6 right); however, isobars and isomers to a certain degree can only be identified by MS^2^ fragmentation. Although low abundant lipids, each TG mass spectrum clearly shows fragments for the neutral loss (NL) of their specific fatty acyl chains (FA) clearly identifying these TG as TG(16:0_18:2_20:4) and TG(18:2_20:5_22:6). These two TGs are not detected in the single THP-1 cells and therefore identified as drivers for the separation of the two cell lines. Identifying their exact acyl chain composition can help future investigations by, e.g., using a stable isotope of FA (20:5) to study THP-1 uptake and phenotype. As with any untargeted lipidomics method, fatty acyl mass overlap due to e.g. isotopic peaks cannot be entirely ruled out.

### Combining Fluorescent Live Cell Imaging and Single-Cell Capillary Sampling with DDA Lipidomics to Study Lipid Droplets after Induced Oxidative Stress

**Figure 4:**
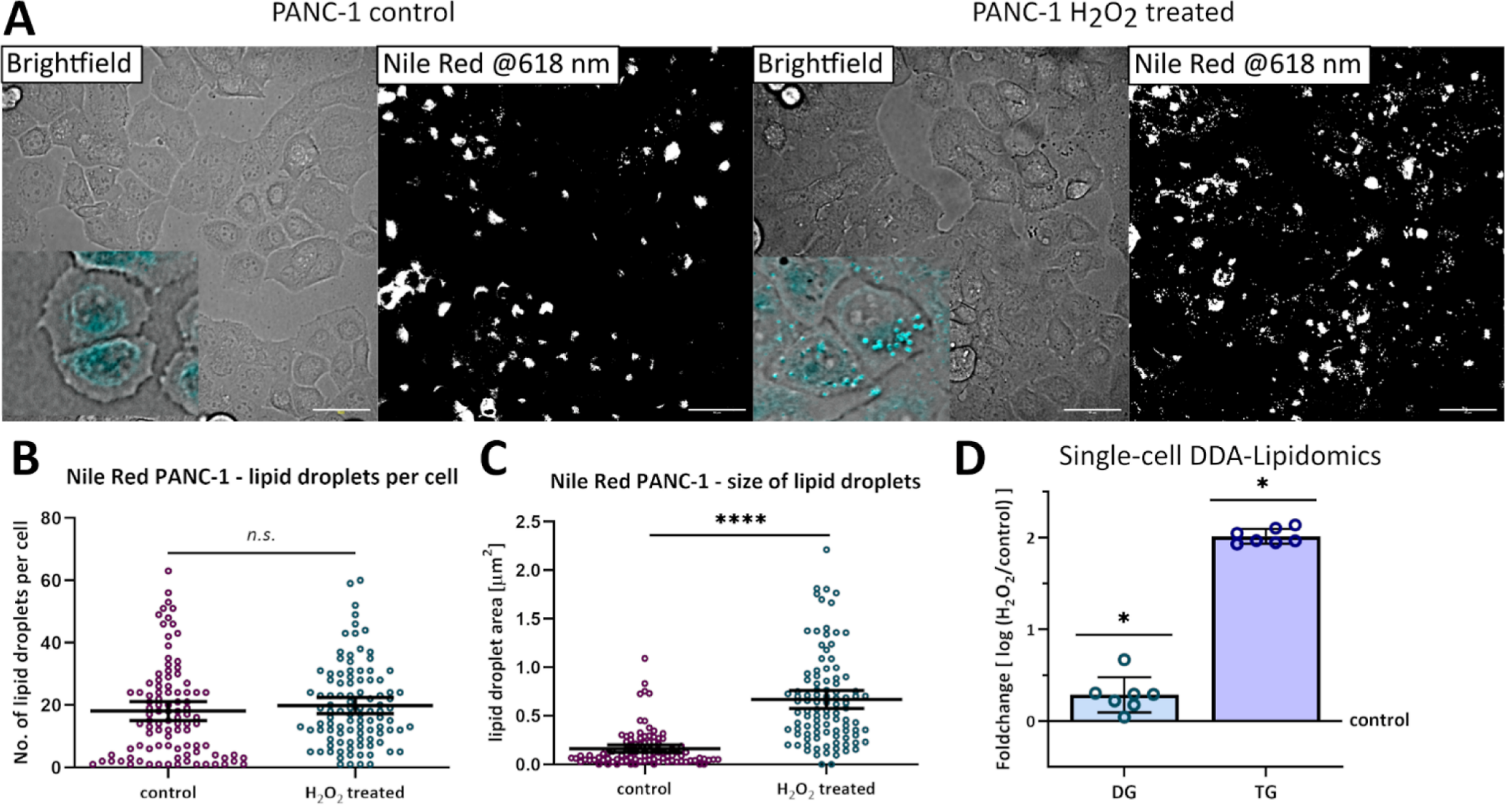
Fluorescent live cell imaging (Nile Red) and single-cell DDA lipidomics of pancreatic cancer cells (PANC-1). (A) Bright-field and fluorescent images of control PANC-1 (left) and H_2_O_2_-treated PANC-1 cells (right; scale bar = 50 μm). Images were taken with the Yokogawa SS2000 Cellome. Nile Red staining of lipid droplets with particle size analysis in ImageJ was used to calculate (B) number of lipid droplets per cell and (C) area [μm^2^] of lipid droplets as an indicator of size (control *n* = 98; H_2_O_2_-treated *n* = 100; unpaired *t*-test *p* < 0.001 for area control vs H_2_O_2_-treated; n.s. = not significant); and (D) fold change as the log of H_2_O_2_-treated vs control lipids for the total detected signal of diacylglycerol (DG; *p* = 0.034) and triacylglycerol (TG; *p* = 0.049; unpaired *t*-test).

PANC-1 pancreatic cancer cells were cultured either untreated (control) or treated with hydrogen peroxide (H_2_O_2_) for 1 h to induce oxidative stress. Lipid droplets were stained with Nile Red for 15 min before being imaged and sampled. Overview images with bright-field and corresponding fluorescent signals (Ex 488 nm/Em 617 ± 73 nm) were taken, as shown in Figure 4A.

The lipid droplet count per cell showed no difference between control and H_2_O_2_-treated PANC-1 cells (control *n* = 98; H_2_O_2_-treated *n* = 100; Figure 4B). However, the size of the lipid droplets measured as area of the fluorescent signal in μm^2^ was four times higher in H_2_O_2_-treated PANC-1 cells compared to control PANC-1 cells (area control = 0.162 ± 0.019 μm^2^ versus area H_2_O_2_-treated = 0.668 ± 0.047 μm^2^ with *p* < 0.001 unpaired *t*-test; Figure 4C). DDA lipidomics was performed on single cells sampled from the same PANC-1 cell culture (same passage number), but lipid profiles were not significantly different according to PLS-DA (control *n* = 11, H_2_O_2_-treated *n* = 7; *R*^2^ = 0.865, *Q*^2^ = 0.217, accuracy = 0.667; data not shown). However, lipid class specific values for the total signal of DG and TG differed significantly when comparing control and H_2_O_2_-treated PANC-1 cells. Total DG lipids detected were 2.1 times (*p* = 0.034) and TG lipids were 1.4 times (*p* = 0.049; unpaired *t*-test) higher in H_2_O_2_-treated PANC-1 cells compared to control cells (Figure 4D). Lipid droplets are intracellular organelles that store neutral lipids and thereby control lipotoxicity in cells, but recent research also proved their contribution to cancer survival and growth.^30,31^ In this study, we simply show the lipid droplet expansion in oxidatively stressed PANC-1 cells through fluorescent imaging and the correlation to increased neutral lipids in living single cells with the methods developed here. We are aware that this correlation is only an indirect one as we are measuring the whole cell and not the lipid droplets. Future work could involve subcellular sampling of lipid droplets to perform lipidomics directly on these organelles. Furthermore, we selected cells for this analysis at random, so future studies could investigate cell selection based on the lipid droplet size and pre-grouping stressed cells to study different phenotypes.

The method presented here opens the possibility to select single cells from mixed cultures based on microscopy information such as phenotype and study direct cell–cell communication and next-neighbor effects in the future. We also showed that the data sets generated by this methodology can be used for the characterization of different cell lines (Figure 3). Developing a method for single-cell analysis will support future research into measuring cell-to-cell heterogeneity within a seemingly homogeneous cell population. Although we mainly use average values to demonstrate the feasibility of our method, in this work there are clear implications for cell heterogeneity, for example, the higher variation in single-cell analysis compared to “1 cell equivalent” injections or the heatmap showing variation within the group of single cells (Figure 2).

The method shows single-cell sampling of adherent cells retaining spatial information combined with high confidence LC–MS/MS (DDA lipidomics) and adequate throughput (15 min LC gradient), as well as the future possibility of high-throughput LC methods.

## Conclusions

We developed a workflow for live cell sampling coupled with microflow LC–MS/MS for single-cell untargeted lipidomics. This proof-of-concept study for single-cell lipidomics has addressed the improvement of confidence in lipid identifications through DDA and MS^2^ database confirmation directly in single cells. The methodology has demonstrated unique capabilities otherwise inaccessible to mass spectrometry imaging because living, single cells were sampled, and fluorescent live cell imaging was used on the same cell culture from which cells were sampled from. The success of this methodology has been demonstrated in its ability to (A) distinguish different cell types based on their lipidome using capillary sampled single cells and (B) make single-cell lipidomic observations consistent with previous observations in the literature based on cell extract analysis.

## Supporting Information Available

Single-cell lipid profile (XLSX)
Intra- and interassay values; DDA-optimized parameters; calibration curve LOD and LOQ; EquiSPLASH standard chromatogram; LOD and LOQ; recovery EquiSPLASH; diluted cell extract from different cell lines; and single-cell analysis of two different cell lines (PDF)
