# Supplementary material for: Single-Cell Untargeted Lipidomics Using Liquid Chromatography and Data-Dependent Acquisition after Live Cell Selection
Source: Anal Chem. 2024 Apr 23;96(18):6922–9. doi: 10.1021/acs.analchem.3c05677 (PMC11079853; doi:10.1021/acs.analchem.3c05677)

Supporting Information:

Untargeted single-cell lipidomics using liquid chromatography and data-dependent acquisition after live cell selection.

Johanna von Gerichten[1‡] and Kyle D. G. Saunders[1‡], Anastasia Kontiza[1], Carla F. Newman[2], George Mayson[3], Dany J.V. Beste[3], Eirini Velliou[4], Anthony D.Whetton[5], Melanie J. Bailey[1*]

1 School of Chemistry and Chemical Engineering, Faculty of Engineering and Physical Sciences, University of Surrey, GU2 7XH Guildford, UK

2 GlaxoSmithKline, Cellular Imaging and Dynamics – Stevenage, SG1 2NY, UK

3 School of Bioscience, Faculty of Health and Medical Sciences, University of Surrey, GU2 7XH Guildford, UK

4 Centre for 3D Models of Health and Disease, University College London, Division of Surgery and Interventional Science, London W1W 7TY, UK

5 vHive, School of Veterinary Medicine, School of Biosciences and Medicine, University of Surrey, Guildford, GU2 7XH, UK

***Correspondence**:
Corresponding Author:
m.bailey@surrey.ac.uk

Table of contents:

Cover page and table of contents S1-2
Figure S 1: Optimising parameters for data-dependent acquisition with the Sciex ZenoTOF 7600 based on 17 µg / mL porcine brain polar lipid standard (Avanti). S3
Equation S 1 Calculations for determination of limit of detection (LOD) and limit of quantification (LOQ). S3
Figure S 2: Determination of limit of detection (LOD) and quantification (LOQ) using EquiSPLASH lipid standard in a 5-point calibration. S4
Figure S 3: Exemplary chromatogram for 16 ng / mL EquiSPLASH lipid standard in 14 cell / µL PANC-1 cell extract using micro-flow liquid chromatography. S4
Table S 1: Intra- and Inter-assay values for EquiSPLASH lipid standard (Avanti). S5
Figure S 4 : EquiSPLASH lipid standard recovery in percent for small volume autosampler injections as 5 µL out of 5 µL compared to 5 µL out of 100 µL. S5
Figure S 5 : Diluted cell extract from three different cell lines. S6
Figure S 6 : Single-cells DDA analysis from two different cell lines S7

Supplementary Figure S 1 Optimising parameters for data-dependent acquisition with the Sciex ZenoTOF 7600 based on 17 µg / mL porcine brain polar lipid standard (Avanti). Values shown are mean ± standard deviation with n = 3. Optimisation was performed for maximum number of lipid features detected.

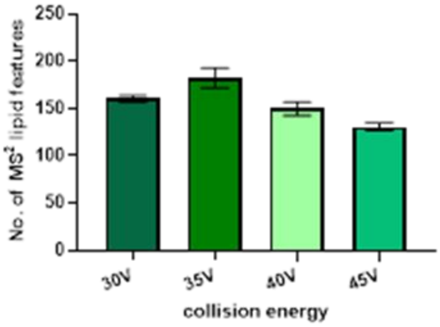

Equation S 1: Calculations for determination of limit of detection (LOD) and limit of quantification (LOQ):

The internal standard (EquiSPLASH) was added to a pancreatic cancer cell (PANC-1) bulk extract of 14 cells/µL in 5 concentrations: 8 , 3.2, 1.6, 1.0 and 0.5 ng/mL as well as 0 ng/mL EquiSPLASH and analysed with the DDA lipidomics method described in the Lipidomics analysis – LC-MS/MS section. Samples were injected as triplicates (n=3) and individual lipid areas exported into GraphPad Prism as XY format (X=concentration; Y=area). A simple linear regression (XY analysis) was performed to determine the slope and Y-intercept for the individual lipids' calibration curve. The LOD and LOQ were then calculated using the following formula:

$$LOD = 3.3 \times (\frac{Y - intercept}{slope})$$

$$LOQ = 10 \times (\frac{Y - intercept}{slope})$$

Supplementary Figure S 2 Determination of limit of detection (LOD) and quantification (LOQ) using EquiSPLASH lipid standard in a 5-point calibration. EquiSPLASH was added to PANC-1 bulk extract (14 cells/µL). Values shown are mean ± standard deviation with n = 3.

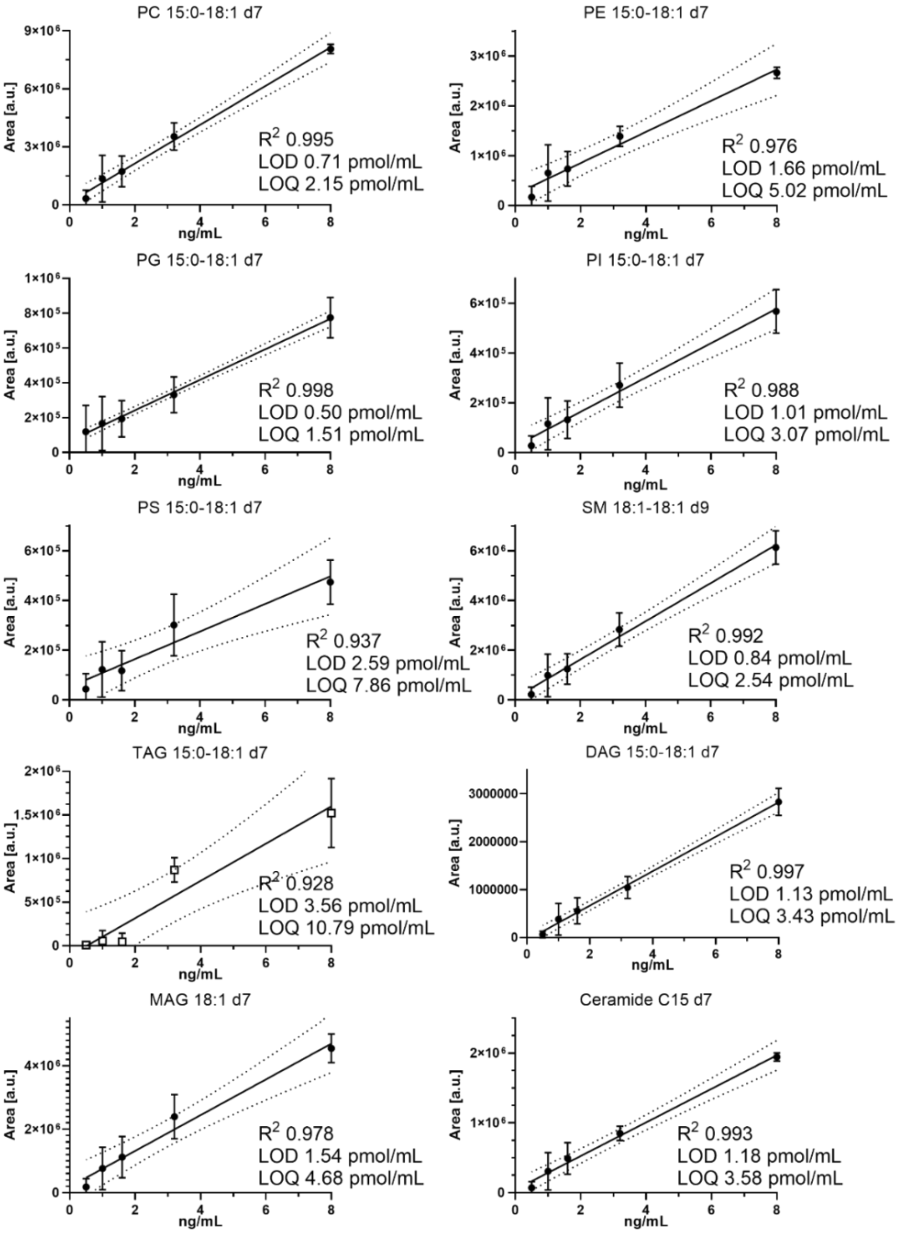

Supplementary Figure S 3 Exemplary chromatogram for 16 ng / mL EquiSPLASH lipid standard in 14 cell / µL PANC-1 cell extract using micro-flow liquid chromatography

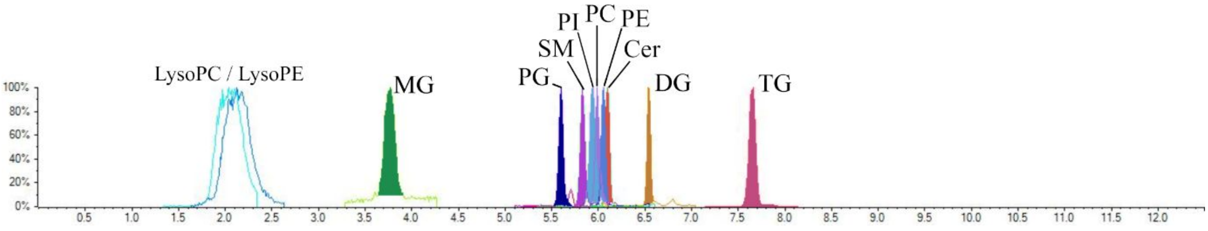

Table S 2: Intra- and Inter-assay values for EquiSPLASH lipid standard (Avanti). Intra-assay EquiSPLASH was injected as 5 µL out of 5 µL (n=4), and Inter-assay as 5 µL out of 100 µL. %CV = is the coefficient of variation. SD = standard deviation.

Lipid	Intra-assay (5 µL) n=4			Inter-assay (high Volume) n=22		
	Mean area	SD	%CV	Mean area	SD	%CV
PC 15:0-18:1 d7	9835248	2408363	24.49	20882124	1540121	7.38
PE 15:0-18:1 d7	2570211	520560	20.25	5413822	344536.7	6.36
PG 15:0-18:1 d7	718428	153489	21.36	1853981	204691.8	11.04
PI 15:0-18:1 d7	477634	108600	22.74	1172193	138873.8	11.85
PS 15:0-18:1 d7	585037	132195	22.60	1851654	129525.9	7.00
SM 18:1-18:1 d9	6637716	1645191	24.79	14994132	810198.5	5.40
TG 15:0-18:1 d7 – 15:0	1202393	314269	26.14	9365493	5218142	55.72
DG 15:0-18:1 d7	3498003	540271	15.45	7276143	536160.3	7.37
Cer C15 d7	1912677	408491	21.36	3669708	191988.1	5.23

Supplementary Figure S 4 EquiSPLASH lipid standard recovery in percent for small volume autosampler injections as 5 µL out of 5 µL compared to 5 µL out of 100 µL (=100%; dotted line). Values shown are mean ± standard deviation with n = 5. Left: autosampler with 5 µL injection loop; Right: autosampler with 10 µL injection loop.

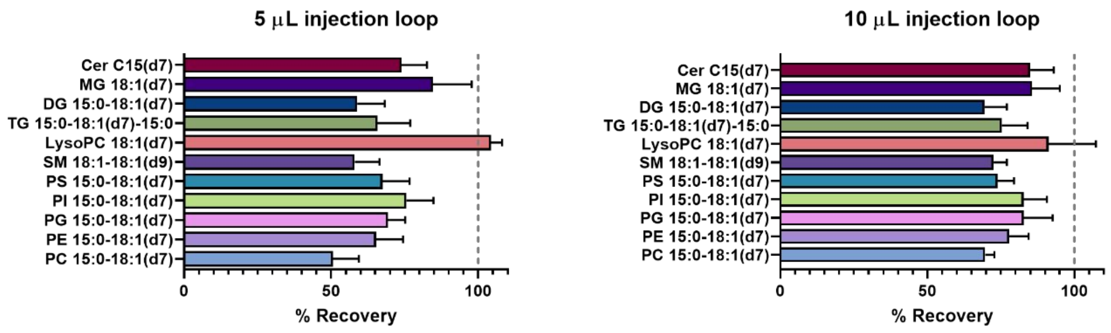

Supplementary Figure S 5 Lipid coverage of diluted cell extract determined with DDA lipidomics as described in Lipidomics analysis – LC-MS/MS. (A) Retention time of phosphatidylcholine (PC) lipid species dependent on carbon chain length (No. of C) and saturation grade (DB = double bond). (B) Change in lipid coverage when analysing different cell types and sizes demonstrating variations up to 85% between cell lines. (n=5; mean±SEM). (C) PLS-DA score plot of three different cell lines showing separation of the groups at 1 cell equivalent injection. (D) VIP plot from PLS-DA analysis of diluted cell extract from three different cell lines (PANC-1, ASPC-1 and THP-1) at 0.2 cells/µL. (n=5).

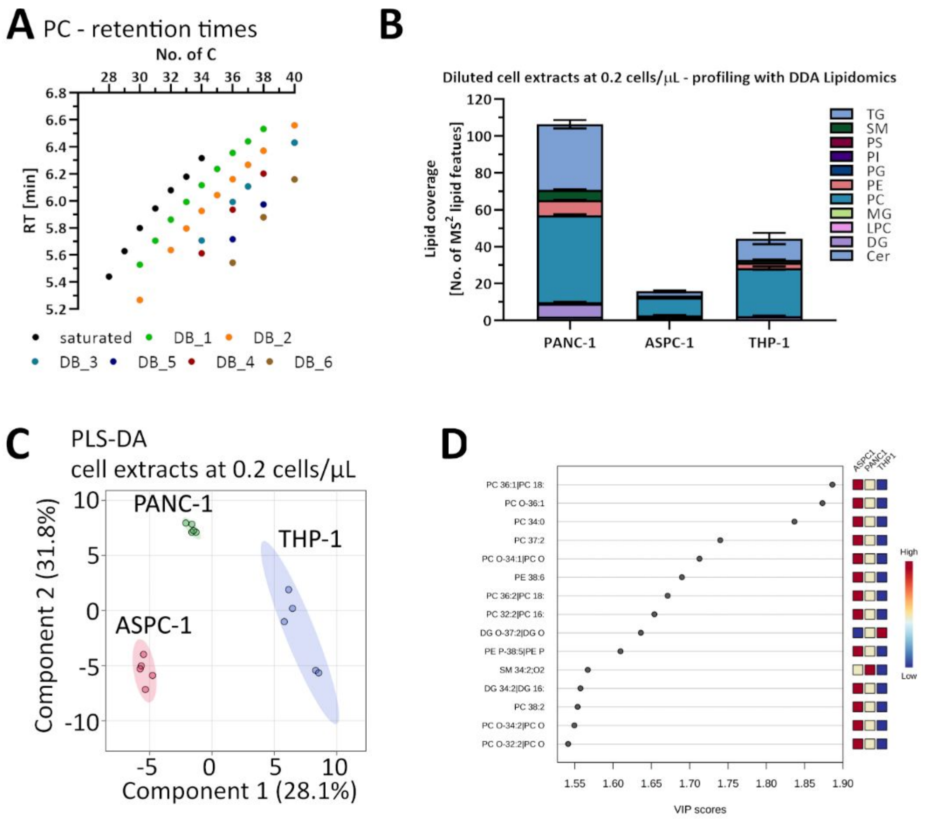

Supplementary Figure S 6 VIP plot (left) from PLS-DA analysis of single-cells from two different cell lines, PANC-1 (n=11) and THP-1 (n=6). The right figure shows the TG lipid species retention time elution in relation to carbon chain length (No. of C) and degree of saturation (DB = double bond) in PANC-1 single cells.

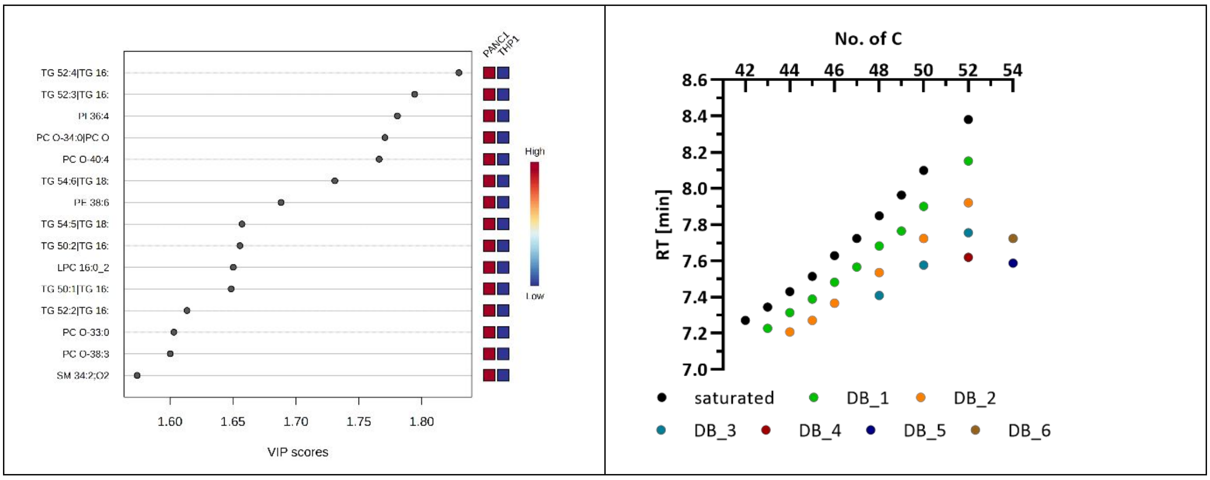

Supplement: Supplementary file 2 — ac3c05677_si_002.pdf [file ac3c05677_si_002.pdf]
